# Molecular Age-Related Changes in the Anterior Segment of the Eye

**DOI:** 10.1155/2017/1295132

**Published:** 2017-09-24

**Authors:** Luis Fernando Hernandez-Zimbron, Rosario Gulias-Cañizo, María F. Golzarri, Blanca Elizabeth Martínez-Báez, Hugo Quiroz-Mercado, Roberto Gonzalez-Salinas

**Affiliations:** ^1^Research Department, Asociación para Evitar la Ceguera “Hospital Luis Sanchez Bulnes”, I.A.P., 04030 México City, Mexico; ^2^Cell Biology Department, Centro de Investigación y de Estudios Avanzados del IPN, 07360 México City, Mexico; ^3^Anterior Segment Surgery Department, Asociación para Evitar la Ceguera “Hospital Luis Sanchez Bulnes”, I.A.P., 04030 México City, Mexico; ^4^University of Colorado, Denver, CO, USA

## Abstract

**Purpose:**

To examine the current knowledge about the age-related processes in the anterior segment of the eye at a biological, clinical, and molecular level.

**Methods:**

We reviewed the available published literature that addresses the aging process of the anterior segment of the eye and its associated molecular and physiological events. We performed a search on PubMed, CINAHL, and Embase using the MeSH terms “eye,” “anterior segment,” and “age.” We generated searches to account for synonyms of these keywords and MESH headings as follows: (1) “Eye” AND “ageing process” OR “anterior segment ageing” and (2) “Anterior segment” AND “ageing process” OR “anterior segment” AND “molecular changes” AND “age.” *Results*. Among the principal causes of age-dependent alterations in the anterior segment of the eye, we found the mutation of the TGF-*β* gene and loss of autophagy in addition to oxidative stress, which contributes to the pathogenesis of degenerative diseases.

**Conclusions:**

In this review, we summarize the current knowledge regarding some of the molecular mechanisms related to aging in the anterior segment of the eye. We also introduce and propose potential roles of autophagy, an important mechanism responsible for maintaining homeostasis and proteostasis under stress conditions in the anterior segment during aging.

## 1. Introduction

According to the Global Health Estimates (WHO 2014) by 2017, for the first time in history, the number of people aged 65 years and older globally will outnumber children younger than 5 years of age (World Health Organization, [1]). Specifically in Latin America (according to the World Population Aging report from the World Health Organization, 2015), over the next 15 years, the number of elderly population is expected to grow faster in this region, with a projected 71 percent increase in the population aged 60 years or over [1, 2].

The anterior segment of the eye comprises all the structures located between the corneal epithelium and the posterior capsule of the lens. This review aims to revise the main molecular, physiological, and age-related changes in the anterior segment of the eye.

## 2. Methods

This review focuses on published articles that address the subject of the aging process in the anterior segment of the eye and the associated molecular and physiological events. We performed a search on PubMed, CINAHL, and Embase for the published literature available using the MeSH terms “eye,” “anterior segment,” and “age.” We used no language restrictions. We generated searches to account for synonyms of these keywords and MESH headings as follows: (1) “Eye” AND “ageing process” OR “anterior segment ageing” and (2) “Anterior segment” AND “ageing process” OR “anterior segment” AND “molecular changes” AND “age”. The search encompassed manuscripts published up to March 2017, and it generated 531 individual references. Abstracts from meetings were not included, as they usually do not contain enough information to perform a proper evaluation. Two researchers (LFHZ and RGS) identified 78 published studies that met the inclusion criteria.

## 3. Results

### 3.1. Primary Age-Related Changes in the Anterior Segment

#### 3.1.1. Ocular Surface and Cornea

Meibomian glands, responsible for the oily component of the tear film, become dysfunctional in most patients aged 60 and older, causing rapid evaporation of the tear film with subsequent dry eye symptoms, discomfort, and visual disturbances [3]. In addition, the cornea suffers changes in its shape and optical properties, including corneal steepening measurable by keratometry and a shift in toricity from with-the-rule to against-the-rule astigmatism and increased collagen interfibrillar spacing, as well as an increased thickness of Descemet's membrane [4]. It also becomes more prone to infections, mainly due to increased epithelial permeability and impaired barrier function secondary to the focal loss of hemidesmosomes that occurs with age [5], as well as decreased phagocytic ability of neutrophils. Within the corneal stroma, the senescent keratocytes overexpress collagenase, stromelysin, and elastase [6]. Increased levels of lipofuscin and endogenous ceramide have been reported. With time, there is an asymptomatic deposition of lipids concentrically to the limbus (cholesterol esters, cholesterol, and neutral glycerides), which is the most frequent age-related corneal change, known as arcus senilis or gerontoxon [7]. Another corneal age-related change is the Hassall-Henle bodies, which consist of localized thickenings in the posterior surface of Descemet's membrane, at the periphery of the cornea, that contain a material thought to be collagen, in which several fissures are filled with extrusions of the corneal endothelium. Although these bodies are present in degenerations and chronic inflammation, they are also associated with the aging cornea [7]. Finally, endothelial cells decrease with increasing age at an annual rate of 0.6%, and since they do not have the ability to regenerate, endothelial cell loss is an important aspect to consider in the aging eye, due to their importance on corneal homeostasis maintenance.

#### 3.1.2. Trabecular Meshwork

As the human eye ages, there are structural changes in the anterior segment of the eye that increase the incidence of glaucoma in this age group. For example, some structural changes that increase the resistance to aqueous outflow occur more commonly in older adults. These changes include thickened trabecular sheets due to accumulation of “curly” collagen and pigment in the trabecular meshwork, decrease of proteoglycans (chondroitin and dermatan sulphates) [8], loss of trabecular endothelial cells, reduction of open pores and spaces of the trabecular meshwork, and accumulation of laminin beneath the endothelial lining of Schlemm's canal [9]. In addition, the loss of trabecular endothelial cells allows the fusion of trabecular beams through hyalinization that results in dysfunctional phagocytosis, as well as macromolecules synthesis and/or degradation that alter physiologic processes.

#### 3.1.3. Lens

Cataracts, the most common cause of vision loss in older people worldwide, are a well-known age-related change. Cataract formation includes the deposition of aggregated proteins in the lens and damage to the plasmatic membrane of lens fiber cells. One of the primary changes during aging is the increase of the relative thickness of the lens' cortex throughout a person's life [10]. This change increases the curvature and therefore the refractive power of the lens, with the concomitant deposition of insoluble particles, which at the same time decreases the refractive index. Therefore, the eye may become more hyperopic or more myopic with age, depending on the predominant change [10].

Chaperone proteins contribute to ensure quality control mechanisms in other to achieve an adequate protein function under normal and stress circumstances [11]. Mitochondria contain two particular chaperones: human heat shock proteins (Hsp) 60 and 70, which protect damaged proteins in the aging eye [12]. The Hsp alpha-crystallin is made of two polypeptides, alpha A crystallin and alpha B crystallin, which are the predominant proteins of the eye lens in vertebrate animals. Alpha A is key for lens transparency, ensuring that alpha B or other close related proteins remain soluble [13]. Nevertheless, as the cell fibers of the lens grow, a proteolytic cleavage of crystallins represents a gradual conversion from water-soluble into water-insoluble proteins. This change induces their aggregation, which in turn provokes subsequent light scattering and lens opacity.

Recently, Augusteyn described a lens increase of 1.3 mg of lens tissue/year during the adult life [14]. However, once polypeptides are synthesized and integrated into mature fiber cells, the capacity to break down proteins using proteases such as caspase presumably persists for some time afterwards to allow organelle degradation [14–16]. Other age-related changes in the lens include decreased concentrations of glutathione and potassium and increased concentrations of sodium and calcium in the cytoplasm of the lens' cells [17].

**Figure 1:**
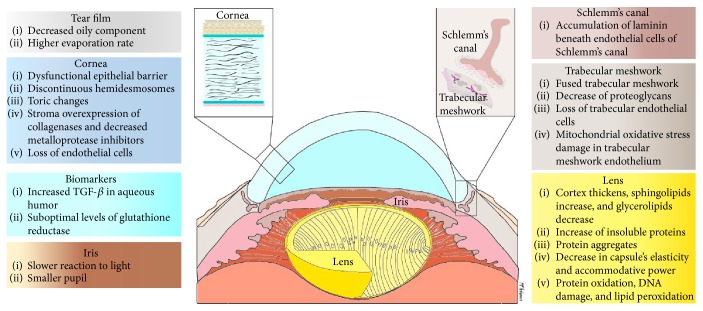
Illustration depicting the most relevant structural changes that occur with aging in the anterior segment of the eye.

All the aforementioned age-related changes in the structures of the anterior segment are depicted in Figure 1.

## 4. Biomarkers

The term biomarker of aging has been defined as a “biological parameter of an organism that either alone or in some multivariate composite will better predict functional capability at some late age than will chronological age” [18].

### 4.1. Inflammatory Markers

Evidence shows that inflammation plays an important role in the aging process; therefore, inflammation biomarkers could be suitable determinants of the aging processes [19]. In the anterior segment of the eye, dysregulation of the complement pathway with altered levels of both intrinsic complement proteins and activated products triggered by oxidative stress has been associated as key players in the aging process [20]. Complement components contribute to pathogenic processes by damaging tissues and being highly chemotactic and capable of facilitating neovascularization [21]. Montalvo et al. recently established an association between C1q, C3, and C4 and corneal and lens anterior capsule damage without inflammation [22], suggesting that molecules released by inflammatory cells and inflamed tissues may affect adjacent tissues not directly involved in the pathogenic process [23].

### 4.2. AGEs

Another cluster of age-related biomarkers is constituted by the advanced glycation end products (AGEs), which are a heterogeneous group of macromolecules formed by nonenzymatic glycation of proteins, lipids, and nucleic acids, where sugars such as glucose react with amino groups in proteins, lipids, and nucleic acids [24]. Aging relates to the presence of AGEs in the cornea, lens, and other ocular structures [24–26]. In the lens, epithelial cell glycation occurs as a reaction of aldo and keto groups of carbohydrates with amino groups of proteins (mostly lysine and arginine residues) [27].

Nevertheless, the lens itself is not only affected by AGEs. Similar to other basal membranes (BM), the lens capsule, a BM secreted by the lens epithelial cells, tends to accumulate posttranslational modifications with age, since the proteins that constitute BMs usually present a low turnover rate [26]. Raghavan et al. reported AGE accumulation in the human lens capsule with increasing age, which in turn is associated with a higher incidence of cataract [28].

In addition, recent studies suggest that AGEs bind to a cell surface receptor known as RAGE. RAGE belongs to the immunoglobulin family of receptors [29]. AGE-RAGE interaction increases intracellular oxidative stress by activation of NADPH-oxidase, a key mediator in superoxide radical production [29]. Therefore, AGEs are linked to another hallmark of aging: oxidative stress.

## 5. Oxidative Stress

Oxidation-reduction mechanisms are of paramount importance in the eye, since oxidative damage can result in specific molecular changes that contribute to the development of age-related sight-threatening diseases such as glaucoma and cataracts.

### 5.1. Reactive Oxygen Species—Reducing Agents

Reactive oxygen species (ROS) comprise a group of molecules formed by the partial reduction of oxygen. They generate in the intracellular space as by-products of cellular aerobic metabolism or may be acquired from exogenous sources due to the exposure of cells to the environment. ROS play an essential role in cell signaling and regulation; however, when their production exceeds the intrinsic antioxidant capacity, they induce damage to cell components such as DNA, proteins, and lipids [30].

The production of ROS, such as hydroxyl radical (-OH), single oxygen (O2), hydrogen peroxide (H2O2), and peroxynitrite (OONO-), has to be balanced with the primary antioxidants and chaperones, reducing agents, antioxidant enzymes, and protein repair systems which protect the tissues against oxidative stress [12]. These reducing systems use electron donors such as glutathione (GSH), NADPH, NADH, FADH2, and thioredoxin.

The main reducing system in the eye is the glutathione system that includes reduced GSH, oxidized glutathione (GSSG), and a number of related enzymes [12, 31]. Glutathione peroxidase reduces H2O2 to water and leads to the oxidation of GSH to GSSG. The reduced state of GSH is maintained by glutathione reductase in which NADPH is needed, hence the importance of glucose-6-phosphate dehydrogenase as well. This system is capable of detoxifying H2O2, dehydroascorbic acid, and lipid peroxides and maintains protein thiols in a reduced state. Other reducing agents include thioltransferase that reduces protein thiols by using reduced GSH [32] and thioredoxin that uses NADPH to maintain mitochondrial proteins in a reduced state [33]. Antioxidant enzymes contribute to the protective role against ROS, like superoxide dismutase, especially SOD2 that converts O2- to hydrogen peroxide. Studies have shown that SOD2 is helpful to protect lens epithelial cells, since cells with high-level expression of this enzyme show resistance to the cytotoxic effects of H2O2, O2-, and UVB radiation [34]. On the contrary, SOD2-deficient cells show mitochondrial damage, leakage of cytochrome C, caspase 3 activation, and increased apoptosis when exposed to O2- [35].

Based on this data, one can conclude that this system is of great significance to ocular and general health maintenance. Therefore, as its efficacy decreases with age, several eye diseases may develop.

### 5.2. ROS in the Lens

As previously mentioned, ROS are generated from intrinsic and extrinsic sources. Through the years, the lens becomes a tissue highly susceptible to oxidative damage since the proteins that constitute it are never replaced. Consequently, protein oxidation, DNA damage, and lipid peroxidation are all found in the process of cataractogenesis [36]. Nevertheless, since the lens has a high concentration of reduced glutathione as previously mentioned, it helps to maintain reduced thiol groups, leading to transparency of the lens and cornea [31].

Phospholipid composition of lens membranes is of particular interest: sphingolipids increase with age, whereas glycerolipids decrease. The decrease in glycerolipids might correlate with the fact that glycerolipids are more prone to oxidation, and at the same time, the growing numbers of oxidized sphingolipids increase membrane stiffness. Studies show that both findings are exacerbated in cataractous lenses [37].

As previously mentioned, concentration of nuclear glutathione (GSH) helps to prevent oxidation. In the lens, epithelial cells are the only ones that accomplish aerobic metabolism and thus the only cells containing mitochondria aside from newly differentiated fiber cells. Damage to these mitochondria leads to a redox imbalance that affects proteins and lipid plasma cell membranes of the fiber cells [30].

The lens has a high metabolic demand mainly in the equatorial regions of the lens epithelium, where cell division and differentiation usually occur [38]. An extensive gap junction network meets metabolic fiber cell demands, from which one can deduce that epithelial cell dysfunction has an important role in lens damage [36]. Recent studies on bovine lenses show metabolically active mitochondria in both epithelial cell and superficial cortical fiber cells [39].

### 5.3. ROS in Glaucoma

Oxidative stress contributes to the pathogenesis of neurodegenerative diseases, including apoptosis of retinal ganglion cells characteristic of glaucoma. The exact molecular physiopathology of POAG is unknown; however, it relates to cellular damage by ROS through direct cytotoxicity and specific amino acid enzymatic oxidation. These protein modifications may lead to glial dysfunction, which spreads neuronal damage by secondary degeneration [40]. Another important structure damaged by oxidative stress is the trabecular meshwork. It has a particular susceptibility to mitochondrial oxidative injury that affects its endothelium. This damage induces cell decay, subclinical inflammation, changes in the extracellular matrix and cytoskeleton, reduced aqueous outflow, and consequently, increased intraocular pressure [41].

## 6. Autophagy and Aging in the Anterior Segment

Cellular homeostasis depends on the proteostasis network that under normal conditions senses and rectifies disturbances in the proteome to restore homeostasis in the cells. Proteostasis maintenance is achieved mainly by two proteolytic systems: the ubiquitin-proteasome and the autophagic system. There are some differences between them: while substrates of the ubiquitin-proteasome pathway are predominantly short-lived proteins, autophagy substrates are long-lived proteins and multiple proteins organized into oligomeric complexes or aggregates not suitable for degradation by other systems [42].

In this regard, autophagy is a catabolic process that “eats” different products (aberrant organelles, misfolded proteins, and protein aggregates) into double membrane autophagosomes and delivers them to lysosomes [43]. The proper function of this process is important because it is the only currently known mechanism that eukaryotic cells possess not only to degrade protein aggregates but also to recycle entire organelles such as mitochondria and peroxisomes [44]. In addition, cell survival is highly dependent on autophagy: loss of autophagy causes accumulation of ubiquitin-positive inclusion bodies and triggers degeneration processes [45].

**Figure 2:**
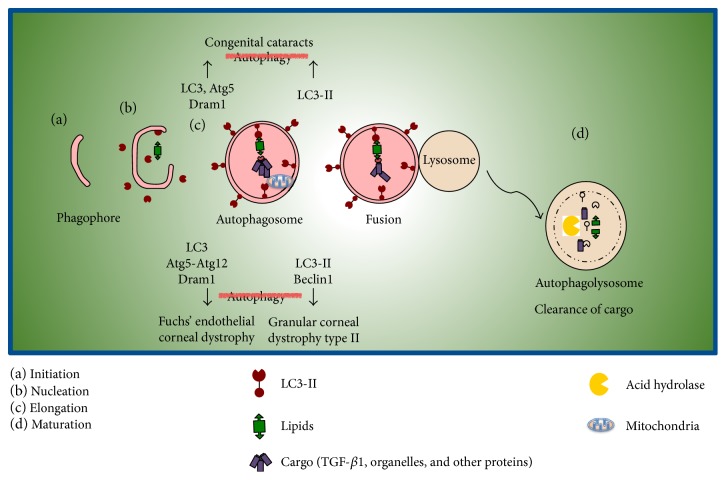
Autophagy elimination of cargo (any protein and/or organelle to be degraded) and autophagy disturbances in the eye are represented in this diagram of corneal and lens cells, displaying autophagy proteins altered in various stages of autophagic processes and the consequences of these modifications.

Although initially autophagy was described as a catabolic process that regulates nutritional homeostasis under stress conditions, currently, autophagy is recognized as a fundamental participant in homeostasis that degrades components that are toxic for the cell. Autophagy is a very complex process and requires a series of coordinated steps. The first step involves the formation of an isolation vesicle called phagophore (Figure 2).

After phagophore formation, it elongates around the cytoplasmic components selected for degradation. The recognition of the components for degradation and the closing of the vesicle are dependent on the lipidated form of LC3 protein (microtubule-associated protein light chain 3). The lipidated form of LC3 is associated with the outer and inner membranes of the autophagosome [46, 47]. A specific pathway that requires at least twenty proteins called ATG (autophagy-related proteins) forms these autophagosomes [47]. Finally, the late stage of autophagy (maturation) depends on the fusion of the autophagosome with the lysosome. This allows contact of the autophagosome cargo with the lysosomal hydrolases and consequently degradation of the components that could be recycled (Figure 2(b)). These steps are fundamental for the autophagic flux (the continuous series of events since the cargo is engulfed until it is degraded). Any event that alters this flux also impairs the degradation process and leads to accumulation of autophagosomes [45–47].

There are some particular stages of the autophagy process. There is an initial stage called initiation that requires a complex formed by the kinase ULK1 (UNC51-like kinase) and its substrates: Atg13 and FIP200. ULK1 may be regulated in two main ways: inhibition by mTOR (target of rapamycin complex 1) and stimulation by AMPK (AMP-activated protein kinase) [47]. During nucleation, the participation of Beclin1 protein, as well as Vsp34 and Atg14, is critical. These proteins form a complex to recruit WIPI1 and Atg2, aiding to form a new autophagosome. Subsequently, elongation and closure of the autophagosome occur. This event involves the formation of an Atg7-dependent conjugated system (Atg12-Atg5), which is responsible for LC3 lipidation by phosphatidylethanolamine. The expansion of this autophagosome membrane is a consequence of the participation of Atg9, and the closure of the autophagosome is a process that helps to include proteins to be degraded (cargo). In the end, cargo degradation is dependent on the interplay between lysosomes and autophagosomes, the so-called autolysosome. One key participant in the transport of autophagic vacuoles is FYCO1 protein [46–47].

In the eye, all cells undergo autophagy in order to maintain a specific and normal function contributing to healthy vision. These cells express differential autophagy-related proteins, but when they harbor gene mutations, they activate stress-induced autophagic pathways and induce the development of ocular diseases [48].

This section of the review summarizes the current knowledge about the role of autophagy in ocular health and disease (specifically cornea and lens), as well as the potential molecules that could be used as a protective therapy against anterior segment degeneration in aging.

### 6.1. Autophagy in Cornea

During aging, there is an overaccumulation of abnormal aggregated proteins in the corneal epithelium and stroma. Among the principal causes of age-dependent accumulation of aggregated proteins in these regions is the mutation of the TGF-*β* gene that affects corneal transparency. This growth factor participates in cell adhesion and migration. In addition, it is recognized as a component of extracellular matrix. The mutant TGF-*β*1 protein is more prone to aggregation, and it is eliminated specifically by autophagy through the interaction between TGF-*β* and LC3. The abnormal accumulation of mutant TGF-*β*1 and the dysregulation of the autophagic process relates to the development of granular corneal dystrophy type II (GCd2) [49, 50]. Indeed, an autophagic inductor suggested for the treatment of GCD2 is lithium. Lithium enhances autophagy by an mTOR-independent pathway, reduces the expression of TGF-*β*1, and increases LC3-II levels [48].

In a mice model of Fuchs' endothelial corneal dystrophy (FECD), the authors observed an increase of LC3 and macroautophagy [49], as well as a decrease in Atg12-Atg5 that affects the complete degradation of different organelles [48].

In addition, in the cornea and conjunctiva, there are infectious (HSV-1 infection) and non-infectious (keratoconus) inhibitors of autophagy, but the mechanisms involved have not been fully understood [48, 51]. The cornea is a target for HSV-1, and after the internalization of the virion and membrane fusion, the viral genome is delivered to the endothelial cell nucleus. As we previously mentioned, Beclin1 is necessary for autophagosome formation through the interaction with Vsp34. In this infection, the virus interacts directly with Beclin1. Some authors have reported different synthetic inductors of autophagy such as the preservative benzalkonium chloride (BAC) [51].

There are several reports describing the participation of autophagy in other pathologies like age-related macular degeneration, diabetic retinopathy (DR), thyroid-associated ophthalmopathy (TAO), chloroquine retinopathy, and glaucoma [52]; however, there are comparatively less reports explaining the autophagy-related mechanisms in the lens.

### 6.2. Autophagy in the Lens

As we mentioned before, during normal aging, the lens losses its clarity and its refractive power diminishes. During maturation of the lens, proteostasis, degradation of the organelles, and nucleic acids produce the organelle free zone (OFZ), contributing to lens transparency. The abnormal growth of lens epithelial cells (LECs) towards the nucleus forms senile cataracts and is a normal process in aging.

Autophagy in the lens normally occurs as a physiologic process to eliminate cytoplasmic components and nucleic acids, indicative of a normal expression of LC3 protein [53, 54]. All the autophagy-related genes and proteins are present in the lens. Some of them, like FYCO1 gene, are involved in lens development and differentiation; also, mutations in FYCO1 gene relate to the development of congenital cataracts [55, 56]. Loss of FYCO1's homeostatic function disrupts the fusion of lysosomes to autophagosomes, resulting in the accumulation of LC3-II vesicles and thus affecting mitophagy [48, 57]. Recently, mitophagy [50] has been extensively studied, but the mechanisms related to nucleophagy and the way they participate in lens transparency with aging have not been fully described [55, 57]. Alterations in some autophagy-related proteins such as Atg5 and Vsp34 are involved in autophagy failure that induces defective lens development, promoting formation of congenital cataracts. However, as we have discussed, there is not enough evidence in the literature to explain thoroughly the autophagic process in lens aging [57].

### 6.3. Autophagy Induction as a Treatment for Anterior Segment Pathologies

A wide variety of molecules can induce autophagy to eliminate accumulated proteins from different cells in the anterior segment. Among autophagy inducers, we find trehalose, metformin, and rapamycin. Trehalose is a disaccharide of glucose (a natural disaccharide that blocks glucose transporters) which “rescues” different products accumulated in corneal endothelium and retina [58]. Several organisms produce it under stress conditions, but it is not a naturally occurring substance in mammals. Specifically in cornea, this sugar suppresses inflammation and neovascularization [58, 59]. In dry eye disease, it helps to decrease cell death as well as inflammation [59]. The literature shows that trehalose prevents neurodegenerative disorders by promoting autophagy, thus reducing the presence of toxic proteins or peptides [59, 60]. Besides, it is not toxic and it can be safely administered in humans [51, 53, 54], like the marketed trehalose eye drops used to preserve viability and function of corneal epithelial cells during desiccation [56, 61]. However, its role in autophagic activation related to anterior segment diseases has not been completely studied.

## 7. Conclusions

Changes in the anterior segment of the eye are responsible for half of the four most common causes of age-related vision impairing diseases (glaucoma, cataracts, age-related macular degeneration, and diabetic retinopathy). The burden of these ocular diseases will not only affect developed countries but also developing regions with limited resources. The structural and molecular changes observed in the anterior eye segment are caused by molecular changes in intercellular unions, structural arrangements of collagen fibers, overexpression of degradation enzymes, underexpression of inhibitors of metalloproteases in tissues, UV light absorbed that produces ROS, inflammatory cytokines and molecules (such as TGF beta), and dysregulation of autophagy, among others. As the global population ages, diseases related to cell and tissue senescence are becoming more prevalent, so it is important to be familiar with these changes in order to tackle their consequences as best as possible.
